# Editorial: Effects of environmental toxins on brain health and development

**DOI:** 10.3389/fnmol.2023.1149776

**Published:** 2023-02-08

**Authors:** Natasha N. Kumar, Yik Lung Chan, Hui Chen, Brian G. Oliver

**Affiliations:** ^1^Faculty of Medicine and Health, School of Biomedical Sciences, University of New South Wales Sydney, Kensington, NSW, Australia; ^2^Faculty of Science, School of Life Sciences, University of Technology Sydney, Ultimo, NSW, Australia

**Keywords:** neuropeptide, neuroscience, maternal smoke exposure, opioid use and pregnancy, air pollutants, chronic obstructive pulmonary disease (COPD)

Exposure to environmental toxins, such as cigarette smoke, polluted air, and pesticides, can negatively impact brain health, leading to cognitive decline and an increased risk of neurodegenerative diseases, such as Alzheimer's Disease. Additionally, exposure to certain toxins during critical periods of brain development can lead to developmental disorders and birth defects. The precise mechanisms by which these toxins affect brain health are yet to be elucidated. Some proposed mechanisms include oxidative stress, inflammation, and changes to neurotransmitters. In this Research Topic, which focuses on the impacts of environmental toxin exposure on brain health, five peer-reviewed papers tackled such research questions from different perspectives addressing toxins coming from natural sources (air pollutants) or human activities (cigarette smoking, opioids). These findings provide new insight into novel potential therapeutic targets and preventative measures to mitigate the detrimental effects on those exposed and their offspring.

Li et al. reviewed existing evidence to identify the mechanisms of by which common air pollutants (PM2.5) induce brain damage. PM2.5 refers to the fine particles in polluted air with a diameter of 2.5 micrometers or smaller. These fine particles can enter the bloodstream and damage the brain *via* various actions, including cerebrovascular damage, brain nerve damage and brain tumor formation. Furthermore, they suggested the underlying mechanisms of action, including autophagy, oxidative stress, neuroinflammation, and autonomic dysfunction. Questions remain as to which brain circuits are involved and whether epigenetic mechanisms play a significant role.

Chronic exposure to tobacco cigarette smoke is a significant risk factor for chronic obstructive pulmonary disease (COPD), the third leading cause of death worldwide (Australian Institute of Health and Welfare, Australian Government, 2020; World Health Organization, 2022). Cigarette smoke contains thousands of toxic ingredients that aggravate airway inflammation, oxidative stress, and lung emphysema. Furthermore, smoking-induced COPD is associated with neurocognitive dysfunction, and brain structural and neurochemical abnormalities; however, the mechanisms remain unknown, and the pathways involved are likely to be complex and multi-factorial. Hypoxia, airway obstruction or/and systemic inflammation are the currently proposed mechanisms (Dodd, 2015), suggesting a significant knowledge gap to be followed up by future studies.

Using an elegant series of behavioral and molecular measures, Dobric et al. demonstrated that mice exposed to cigarette smoke for 6 months exhibit a COPD phenotype accompanied by neurocognitive dysfunction, modeling human patients. Alongside multiple measures detecting lung pathology, the authors found that the tight junctional protein Zonula occludens (ZO)-1 was decreased in cigarette smoke exposed mice compared to sham controls. This supports existing evidence of a compromised blood brain barrier initiating brain damage in those with smoking-induced COPD. They also demonstrated the development of microglia-dependent neuroinflammation in the hippocampus due to chronic cigarette smoke exposure. This could provide a framework for future clinical interventions to protect the brain from neuroinflammatory sequalae in patients with COPD.

Smoking can also interfere with post-surgical recovery, causing smokers to suffer from post-surgical complications more frequently than non-smokers, including impaired heart and lung functions, wound healing, and anesthetic recovery. Wang et al. explored the association of cigarette smoking with post-operative pain by examining cerebrospinal fluid *(*CSF) levels of the neuropeptides beta-endorphin and substance P in a Chinese cohort. Compared to non-smoking controls, active smokers exhibited higher subjective pain intensity and longer post-surgical recovery time accompanied by lower beta-endorphin and higher substance P levels in the CSF. The findings suggest that interventions to increase beta-endorphin and reduce substance P levels in the CSF of active smokers may be effective in reducing post-operative pain intensity. Substance P is a sensory neurotransmitter important in the perception of pain (Douglas and Leeman, 2011). To target this mechanism, tachykinin receptor 1 (NK1R) antagonists can cross the blood brain barrier to attenuate nociceptive responses to inflammation or nerve damage in animal models of pain; however, unfortunately there is little evidence of its analgesic efficacy in humans (Pintér et al., 2014; Yang et al., 2021). Nevertheless, there is hope that novel NK1R antagonists (e.g., aprepitant) could one day be repurposed as a therapy to limit neuroinflammation-induced post-operative pain. With advances in ultrasensitive detection systems, future patients could benefit from the measurement of substance P and beta-endorphin in blood samples, allowing for less invasive screening for pain or neuroinflammation.

Maternal smoking during pregnancy negatively affects genetically programmed brain development in the offspring, because of carbon monoxide, free radicals, nicotine and other toxic chemicals (Dempsey and Benowitz, 2001; Ekblad et al., 2015). Detrimental outcomes in childhood associated with tobacco smoke exposure *in utero* include congenital heart disease, respiratory and allergic sequela, malignancy and neuropsychiatric disorders (Maciag et al., 2022). Using MRI image analysis, Ekblad et al. report that when brain volume is proportional to intracranial volume, maternal smoking during pregnancy is associated with smaller gray matter volumes in adolescent offspring, suggesting that these defects may contribute to the development of neuropsychiatric disorders.

Like smoking, maternal exposure to opioids can also have adverse and long-lasting outcomes on the developing human brain. Due to the opioid epidemic, the Schedule III opioid analgesic buprenorphine is now preferentially used for treating pregnant patients with opioid use disorder. Buprenorphine is clinically indicated as a first line treatment over other opioids, such as oxycodone and fentanyl, due to its safer therapeutic index, effectiveness, and favorable outcomes for both mother and offspring (Maciag et al., 2022). Nieto-Estévez et al. used rodent and organoid models of early life exposure to investigate acute and long-term consequences of buprenorphine exposure on the developing brain. Results from *in vivo* spheroid cell cultures demonstrate that μ-, κ- or δ-opioid receptors are absent, and the nociceptin opioid peptide (NOP) receptor is abundant in the developing human brain. Although buprenorphine was found to convey inhibitory signals *via* the NOP receptor and alter gene expression and interneuron migration into the cortex, neural activities were not changed. In rats, the authors demonstrated that prenatal exposure to buprenorphine alters interneuron migration in the adult cortex. These findings may implicate buprenorphine exposure *in utero* in the etiology of neurological diseases arising from disrupted interneuron function in the cortex (e.g., autism, schizophrenia), especially given that buprenorphine has modest NOP receptor agonist activity. Future investigation of the pharmacological interactions between NOP receptor antagonists or NOP receptor knock out animals, in the context of prenatal buprenorphine exposure, will continue to give essential information about the influence of buprenorphine on neural circuits involved in psychiatric illness.

The articles published in this Research Topic highlight cigarette smoking, maternal cigarette smoking, opioids use during pregnancy, and PM2.5 as environmental toxins contributing to the pathogenesis and etiology of brain dysfunction. These studies advance our understanding of the molecular mechanisms involved in acute and long-term exposure to environmental toxins.

## Author contributions

NK prepared an initial draft of this editorial. It was carefully revised by HC. All authors approved the final version for submission.
